# Impact of Oxygen Stoichiometry on the Thermoelectric Properties of Bi_2_Sr_2_Co_2_O_*y*_ Thin Films

**DOI:** 10.1021/acsaem.4c00551

**Published:** 2024-05-14

**Authors:** Arindom Chatterjee, Alexandros El Sachat, Clivia M. Sotomayor Torres, José Santiso, Emigdio Chavez-Angel

**Affiliations:** † Catalan Institute of Nanoscience and Nanotechnology (ICN2), CSIC, Barcelona Institute of Science and Technology (BIST), Bellaterra 08193, Spain; ‡ Institute of Nanoscience and Nanotechnology, National Center for Scientific Research “Demokritos”, Athens 15341, Greece; § ICREA—Catalan Institute for Research and Advanced Studies, Barcelona 08010, Spain

**Keywords:** thermoelectrics, misfit cobaltates, oxygen annealing, Bi_2_Sr_2_Co_2_O_*y*_, spin−orbit degeneracy, power factor

## Abstract

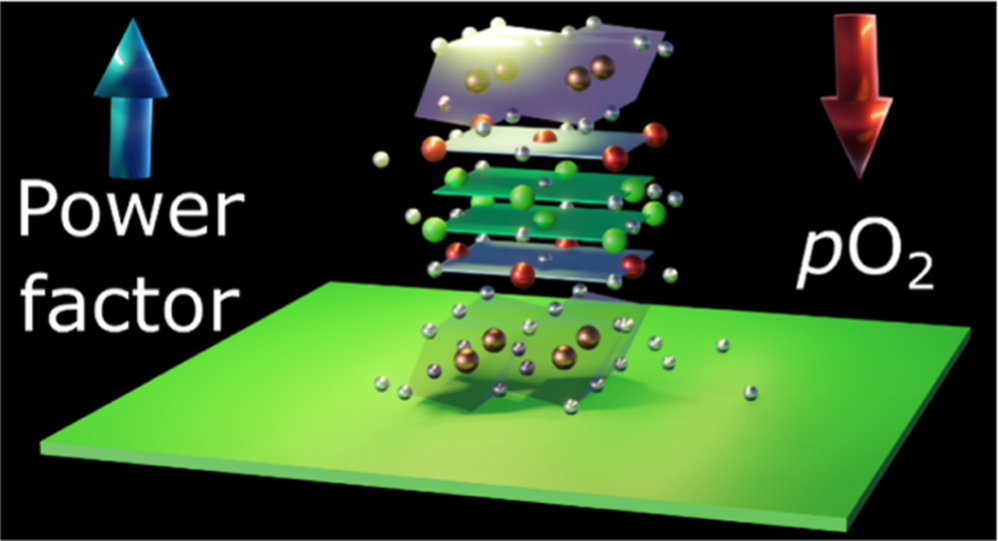

In this study, we present a comprehensive analysis of the thermoelectric (TE) properties of highly *c*-axis-oriented thin films of layered misfit cobaltates Bi_2_Sr_2_Co_2_O_*y*_. The films exhibit a high *c*-axis orientation, facilitating precise measurements of electronic transport and TE properties along the *a*–*b* crystallographic plane. Our findings reveal that the presence of nearly stoichiometric oxygen content results in high thermopower with metallic conductivity, while the annealing of the films in a reduced oxygen atmosphere eliminates their metallic behavior. According to the well-established Heike’s limit, the thermopower tends to become temperature independent when the thermal energy significantly exceeds the bandwidth, which provides a rough estimation of charge carrier density by using the Heike’s formula. This observation suggests that the dominant contribution to the thermopower comes from the narrow Co–t_2g_ bands near the Fermi energy. Our study demonstrates that the calculated thermopower value using Heike’s formula, based on the Hall electron density of the Bi_2_Sr_2_Co_2_O_*y*_ thin films at 300 K, aligns well with the experimental results, shedding light on the intriguing TE properties of this family of layered cobaltate oxide films.

## Introduction

Layered materials offer significant potential for the development of highly efficient thermoelectric (TE) materials due to their directional TE properties, such as anisotropic thermal conductivity,^1−3^ and high in-plane TE power factor.^4−6^ In particular, layered cobaltates (e.g., Na_*x*_CoO_2_, Ca_3_Co_4_O_9_, Bi_2_Sr_2_Co_2_O_*y*_, and La_2–*x*_Sr_*x*_CoO_4_) have emerged as promising p-type materials for TE application due to (i) their unique combination of electronic and thermal properties,^7−10^ (ii) earth abundance of their constituent elements, (iii) high temperature stability, and (iv) the nontoxic nature of their constituent elements compared to commercial materials, e.g., Bi_2_Te_3_. These materials exhibit complex behaviors arising from the interplay of charge, spin, and lattice vibrations, making them highly versatile for energy conversion applications.^7,11,12^ A worth noting advantage of cobaltates is their intrinsic high in-plane electrical conductivity (σ), coupled with low thermal conductivity (*k*) and high Seebeck coefficient (*S*),^13−21^ enabling them to produce significant voltage differences when exposed to temperature gradients. An additional advantage lies in the particular misfit crystal structure of this class of layered cobaltates, which results in anisotropic thermal properties with low in-plane (*k*_∥_ = 6–1.5 W m^–1^ K^–1^)^22−25^ and out-of-plane (*k*_⊥_ = 1–0.07 W m^–1^ K^–1^)^23−26^ thermal conductivities. Consequently, this attribute enhances the overall TE figure of merit, *zT* > 1,^20,25,27^ where *zT* = *S*^2^·σ·*T*/*k* or PF/*k*, where σ represents the electrical conductivity, *T* the average temperature, and PF the TE power factor (*S*^2^·σ) multiplied by the average temperature (PF = *S*^2^·σ·*T*). Moreover, Paul et al. showed that the introduction of a disordered nanoporosity presents a viable strategy to further enhance the TE properties of Ca_3_Co_4_O_9_.^28,29^ Through selective scattering of phonons (the main heat carriers), they managed to reduce the k value without significantly affecting the electronic transport. Similarly, Xiao et al. observed analogous phenomena in MoS_2_ suspended membranes.^30^ Their findings indicated that periodic arrays of holes, even with periods exceeding the average phonon mean free path, significantly decrease the k value with a relatively small disruption of σ.

**Figure 1:**
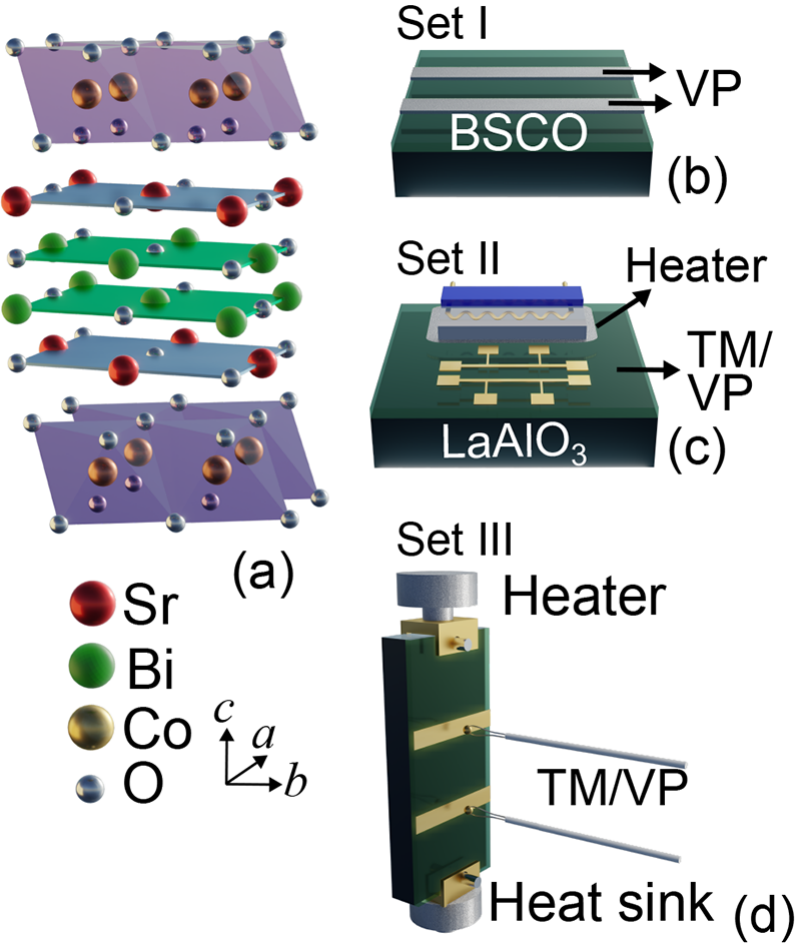
Schematic representation of the sample structure and experimental setup for measuring the TE properties. (a) Crystallographic structure of Bi_2_Sr_2_Co_2_O_9_ adapted from ref (44). (b) Sample for ac measurements. (c) Homemade Seebeck coefficient setup: the metallic stripes deposited on top were used as thermometers (TM) and voltage pads (VP) for measuring the Seebeck voltage created by the ceramic heater. (d) Commercial setup (LINSEIS) for measuring the Seebeck effect and electrical conductivity.

The misfit crystal structure consists of sequential layers along the *c*-axis of two subunits possessing different crystallographic symmetries, thereby exhibiting an incommensurate crystal structure. For the case of Bi_2_Sr_2_Co_2_O_*y*_ (BSCO) compound, one subunit encompasses hexagonal CoO_2_ layers, wherein the cobalt oxide octahedra shares their edges, while the other subunit shows a highly ordered rock salt (RS) structure consisting of Bi_2_Sr_2_O_4−δ_ layers. These subunits grow along the crystallographic *c*-axis but display a considerable mismatch along the *b*-axis, hence the term “misfit” (see Figure 1a). Specifically, the *b*-parameter of the hexagonal layer (*b*_hex_) is shorter than that of the RS layer (*b*_RS_). Their ratio is defined as the misfit ratio, *q*.^31^ In general, the crystal structure of BSCO is presented as [Bi_2_Sr_2_O_4−δ_]_RS_[CoO_2_]_*q*_.^32^ BSCO single crystals exhibit promising TE properties, which can only be achieved in a particular crystallographic direction (in-plane); thus, their TE properties are highly anisotropic.^33,34^ Consequently, the TE properties of polycrystalline misfit cobaltates face significant reduction due to the random orientation of the crystallites, as well as the grain boundary contribution.^17,35−39^

To overcome this limitation and explore the anisotropic electronic and TE properties in specific crystallographic directions, researchers have turned to the epitaxial growth of thin films of these layered cobaltates, which allows us to (i) achieve crystalline quality of thin films close to a single crystal and (ii) control growth orientations and, hence, physical properties along the particular crystallographic axis. However, the growth of epitaxial thin films of misfit cobaltates using physical vapor deposition techniques has proven to be challenging. Ravichandran et al. showed that the first few unit cells of BSCO films grown on the yttria-stabilized zirconia substrate exhibited electrical insulating behavior.^40^ Despite progress in growing highly *c*-axis oriented films, in-plane textures have exhibited either random oriented^40^ or, in some cases, partial ordering.^41^ The term “fiber texture” is specifically used when a film has a single out-of-plane orientation with randomly oriented crystals in the in-plane direction. This indicates the complexity associated with obtaining a perfect epitaxial film of misfit cobaltates. However, in-plane epitaxy of misfit thin films have been achieved by chemical solution-^42^ and reactive solid-phase epitaxy methods.^43^

A common approach to control and improve the growth of oxide-based thin films is through oxygen stoichiometry manipulation. This approach is also used to modify the electronic properties of transition-metal-based oxides, including both bulk materials and films. In the case of BSCO single crystals, notable sensitivity of metallic conductivity to variations in oxygen stoichiometry has been observed.^44^ Remarkably, annealing of bulk crystals in an Ar atmosphere at 773 K induces a transition in the temperature dependence of σ from metallic-like to semiconductor-like behavior.^45^ This effect extends to BSCO thin films as well, where an increase of oxygen content in the films yields a substantial enhancement in the transverse TE voltage.^46^ Consequently, the investigation of the impact of annealing on the TE properties of BSCO thin films is very important.

In this study, we investigate the influence of oxygen stoichiometry variations on the in-plane conductivity of BSCO thin films and observe its impact on the TE power factor. Our findings reveal a remarkable sensitivity of the metallic conductivity in BSCO thin films to changes in oxygen stoichiometry. However, intriguingly, we observe that despite these variations in conductivity, the TE power factor remains relatively stable. This observation highlights the decoupling between the metallic conductivity and the TE performance of BSCO thin films, suggesting that other factors may dominate the TE characteristics.

Highly *c*-axis-oriented good-quality BSCO thin films were grown on (001) LaAlO_3_ (LAO) substrates with no preferential in-plane orientations by the pulsed laser deposition (PLD) technique. Thermal annealing of these thin films under different gas atmospheres exhibited a remarkable change in their in-plane electronic conductivity and TE properties. A transition from metallic-like conductivity to a semiconductor-like behavior is observed. On the other hand, the temperature dependence and magnitude of thermopower in the BSCO thin films closely resemble those observed in single crystals. The magnitude of thermopower is impacted by annealing, but its temperature dependence remained almost the same. Furthermore, our data analysis suggests a close agreement between the measured thermopower of the thin films at around 300 K and the predicted thermopower derived from Heike’s formula.^47^

## Experimental Methods

200 nm-thick BSCO films were deposited on (001) LAO single-crystal substrates (5 × 5 × 0.5 mm^3^, Crystec GmbH) using the PLD technique. The growth process occurred at 973 K using a KrF excimer laser (248 nm) with a pulse repetition rate of 3 Hz and a fluency of 1.8 J/cm^2^. A nominally stoichiometric dense ceramic target was used during the deposition, ensuring the appropriate composition of the films.^16^ Throughout the deposition, a stable dynamic oxygen pressure (*p*O_2_) of 150 mTorr was maintained. Subsequently, during the cooling process at a rate of 20 K/min, *p*O_2_ was increased to 600 mTorr to ensure an optimal oxidation state of the Co ions. Structural characterization, including analysis of orientation and in-plane texture, was performed using X-ray diffraction techniques using a Panalytical X’pert Pro MRD diffractometer. The TE measurements were conducted using both a custom-made approach (see Figure 1c) and a commercial system (LINSEIS instrument, Germany; see Figure 1d). Transport properties of BSCO films on LAO substrates were measured on three sets of samples: set I, high-temperature ac electronic conductivity measurements in a variable *p*O_2_ atmosphere; set II, low-temperature TE transport properties between 40 and 300 K in a home-built system; and set III, high-temperature TE transport (325–500 K) in a LINSEIS instrument.

In sets II and III, three samples were measured: one in its original as-grown condition, while the other two underwent annealing. One sample was annealed under a pressure of 1 atm *p*O_2_, referred to as “high O_2_”, and the other at 2 × 10^–3^ atm *p*O_2_, denoted as “low O_2_”, both annealed at 870 K. Oxygen pressure reduction was achieved by diluting pure oxygen with nitrogen gas.

In set I, high-temperature ac electrical conductivity (σ_ac_) measurements were conducted between *T* = 300 and 850 K using a 2-contact geometry and a 1 kHz frequency (see Figure 1b). Set II included low-temperature (40–330 K) transport property measurements for three samples: as-grown, high, and low O_2_ annealed. Initially, electronic conductivity and Hall effect measurements were performed in van der Pauw geometry with magnetic fields up to 1.2 T applied perpendicularly to the film surface, incrementing in 0.2 T steps.

Following the electronic conductivity and Hall measurements, two Pt stripes were deposited using an electron beam metal evaporator and designed via optical lithography on the surface of films (see Figure 1c). The Pt stripes were used as thermometers (TM) and voltage pads (VP) for Seebeck effect measurements. A 100 Ω resistor (Tru components 1583791) was glued on the surface of the film by a thermal paste and used as a heater. The resistance of the two Pt stripes was calibrated as a function of cryostat temperature. A photograph of the device is shown in Figure S2 in the Supporting Information. A detailed description of the methodology is available within the Supporting Information of refs (48 and 49). Later on, a series of thermal gradients were created by applying variable current to the heater at a fixed cryostat temperature, and corresponding Seebeck voltages were measured. As the source of heating is Joule effect, Δ*T*s are proportional to the applied power (*I*^2^*R*) to the heater. *S* was calculated from the slope of the −Δ*V* vs Δ*T* curve (see Figure S1 in the Supporting Information). All the measurements were carried out under vacuum conditions.

**Table 1:** Sample Summary

sample set	purpose of the sample	posterior annealing	annealing conditions	sample’s name
set I	high-temperature XRD and ac electrical conductivity measurements (300–850 K)	in situ, during experiments	as grown	set I: as grown
set II	low-temperature TE measurements (40–330 K)	yes	as grown	set II: as grown
			*T* = 870 K^a^	set II: low O_2_
			*P* = 2 × 10^–3^ atm^b^*p*O_2_	
			*T* = 870 K	set II: high O_2_
			*P* = 1 atm *p*O_2_	
set III	high-temperature TE measurements (325–550 K)	yes	as grown	set III: as grown
			*T* = 870 K	set III: low O_2_
			*P* = 2 × 10^–3^ atm *p*O_2_	
			*T* = 870 K	set III: high O_2_
			*P* = 1 atm *p*O_2_	

a *T* refers to annealing temperature.

b *P* is the annealing gas pressure.

Set III samples were grown on a 10 × 5 × 0.5 mm^3^ (001) LAO substrate for the simultaneous electrical conductivity and Seebeck coefficient measurements in the LINSEIS instrument, from 325 to 550 K. Two 50 nm-thick gold stripes were deposited on the surface of the film to achieve good electrical contact with the thermocouples (see Figure 1d). A summary of the studied samples is shown in Table 1.

## Results

**Figure 2:**
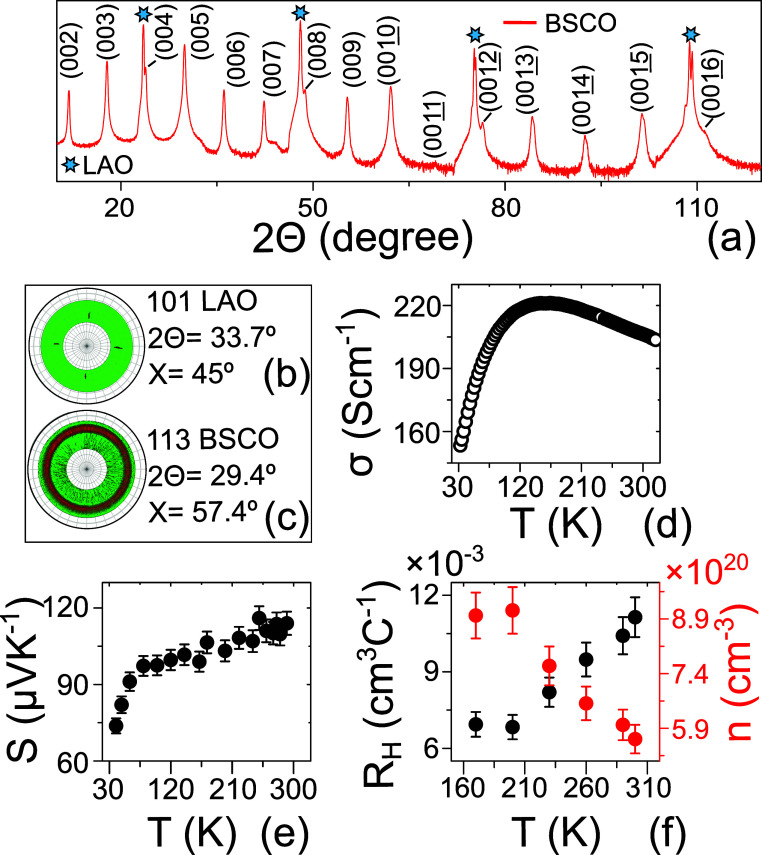
Structural and transport characterization of as-grown Bi_2_Sr_2_Co_2_O_*y*_ thin films: (a) standard X-ray diffraction patterns. The X-ray pole figure is around (b) 101 reflection of LAO substrate and (c) 113 reflections of the Bi_2_Sr_2_Co_2_O_*y*_ thin film. Temperature dependence of (d) electrical conductivity, (e) Seebek coefficient and (f) Hall coefficient and carrier density.

Figure 2a shows the conventional 2θ/ω XRD patterns of a 200 nm-thick BSCO film grown on a LAO substrate. The XRD pattern depicts that film reflections originate from the (0 0 *l*) crystallographic planes of BSCO, corresponding to an out-of-plane lattice parameter of 14.928 Å, determined with a precision of ±10^–4^ Å. This finding indicates that the film is highly oriented along the *c*-axis. The in-plane texture of the film was determined by X-ray pole figure measurements as shown in Figure 2b,c. The stereographic projection of the plane orientation distribution is depicted for 101 and 113 reflections for LAO (Figure 2b) and BSCO (Figure 2c), respectively. The 101 reflection of the substrate exhibited four distinct reflections separated by 90° in azimuthal angle (φ), while maintaining a constant χ angle of 45°. This behavior is indicative of a pseudocubic structure with 4-fold symmetry, as expected. Conversely, the 113 reflection of the BSCO film exhibited a continuous distribution in φ, forming a ring in the stereographic projection. This suggests that the BSCO film does not exhibit any preferred in-plane (*ab*-) orientations. It is important to note that while the film does not display complete epitaxial alignment with the substrate, it does exhibit a pronounced orientation along the *c*-axis direction, which indicates that the layered structure of the film is preserved, and the fiber texture of the film is achieved.

The transport properties of the as-grown BSCO film, within the low-temperature range of 40–350 K, are shown in Figure 2d–f. At room temperature, σ exhibits a value of approximately 210 S cm^−1^. Conductivity was measured with high accuracy (error bars of less than 1%). As the temperature decreases from 330 to 150 K, a characteristic metallic behavior is observed. However, a downturn in conductivity is noted as the temperature descends below 150 K (see Figure 2d). It is worth mentioning that, in the case of single crystals of BSCO, the minimum of the σ–*T* curve appears around *T* = 80 K.^33,50^ Experimental measurements of the electronic band structure at lower temperatures suggest the presence of metallic ground states,^51^ but the downturn of the electrical conductivity is attributed to the formation of a pseudogap.^32,51^ At room temperature, the measured Hall coefficient is *R*_H_ ∼ 0.011 cm^3^ C^–1^ (see Figure 2f). Electron density *n* = 1/(*R*_H_·*e*) (where *e* is the electron charge) was estimated to be *n* = 5.6 × 10^20^ cm^–3^ at 300 K. It showed a linear decrease with temperature from 300 to 150 K in the BSCO film.

At 300 K, measured *S* registered a value of ∼110 μV K^–1^. It remained almost temperature independent from 250 to 300 K, indicating that the electron bands near Fermi energy are narrow. From 250 to 80 K, *S* exhibited a gradual decrease, followed by a sharp reduction below 80 K (see Figure 2e). Notably, the magnitudes of σ, *R*_H_, and *S*, along with their respective temperature dependencies, closely resembled those of their bulk single-crystal counterparts.^33,50^ This is an indication of the high crystal quality of the BSCO thin films obtained by the PLD technique.

To investigate the impact of annealing on the TE properties, thin films of BSCO were subjected to annealing at 873 K under variable *p*O_2_ conditions. Before proceeding with the annealing process, the structural stability of the film was tested through temperature-dependent XRD measurements. The sample was placed in a synthetic air atmosphere and heated from 298 to 773 K and then cooled to room temperature again.

**Figure 3:**
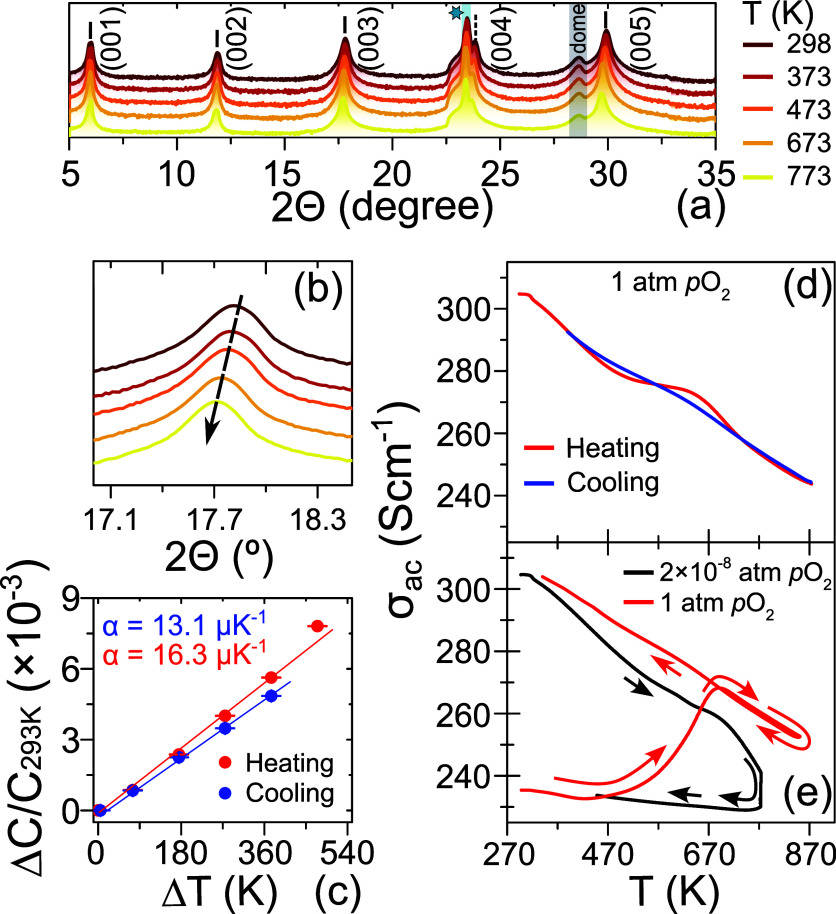
High-temperature crystallographic and transport characterization of the Bi_2_Sr_2_Co_2_O_*y*_ film. (a) Selected portion of the 2θ–ω XRD pattern from 5 to 35° in 2θ. The peaks marked with a cyan start and black rectangle correspond to the substrate and graphite dome used in the experiment, respectively. (b) Lower angle shift of the 003 film reflections in 2θ with increasing temperature from room temperature to 773 K. This indicates the thermal expansion of the BSCO film along the *c*-axis direction. (c) Extraction of the thermal linear expansion coefficient (α) of the BSCO film from the heating–cooling data. High-temperature (350–873 K) ac electrical conductivity measurements of set I film in a controlled oxygen partial pressure atmosphere: (d) heating–cooling cycle of ac electrical conductivity when 1 atm pO_2_ was maintained and (e) electrical conductivity response upon reduction and oxidation.

Figure 3a and b depict the XRD patterns of the BSCO thin film at different temperatures. All film reflections can be indexed with 00*l* Miller indices, and no additional peaks were found in the XRD patterns. This indicates the absence of secondary phases or domain with orientations different from the *c*-axis direction. Additionally, we observe a gradual shift of the 003 peak toward lower in 2θ angles with increasing temperature, suggesting a linear thermal expansion of the *c*-parameters from 14.921 to 15.038 Å (see Figure 3b). The slopes of the heating–cooling curves are slightly different from each other, and the room-temperature *c*-parameter was slightly shorter after cooling than the as-grown film. This could be because of a slight deviation of oxygen stoichiometries before and after heating. From these data, an average thermal expansion coefficient (α) was extracted, yielding an average value of α ∼ 1.47 × 10^–5^ K^–1^ in good agreement with that reported in the literature (see Figure 3c).^52,53^ Overall, the heating–cooling process remains reversible within the temperature range from room temperature to 773 K.

To assess the instantaneous response of the thin film when subjected to specific gas pressure at elevated temperatures ranging from 350 to 870 K, real-time ac electronic conductivity (σ_ac_) measurements were performed at a frequency of 1 kHz (see Figure 3d and e). When maintaining a constant *p*O_2_ of ∼1 atm, σ_ac_ exhibited high stability and reversibility throughout the heating and cooling cycles (see Figure 3d). The linear decrease in σ_ac_ with increasing temperature within the range of 350–850 K suggests a metallic-like behavior of the film. Conversely, the heating–cooling cycle of σ_ac_ became irreversible when the film was exposed to a *p*O_2_ of 2 × 10^–3^ atm (a gas mixture of 79% N_2_ and 21% O_2_) within the same temperature range (see Figure 3e), typical of a loss of oxygen stoichiometry in the film. The distinct slopes in the linear σ_ac_–*T* curve between the heating and cooling cycles suggest that the film, after exposure to low O_2_ annealing, may have lost its metallic characteristics. However, following reannealing (red curve in Figure 3e) under a *p*O_2_ of ∼1 atm, the film recovered its original σ_ac_ value and its linear temperature dependence. This oxidation–reduction behavior displays a high degree of reversibility and closely parallels the observed variations in oxygen stoichiometry, as evidenced by thermogravimetric experiments conducted on bulk Ca_3_Co_4_O_9_ crystals.^54^ By comparing these findings, it can be reasonably argued that the changes in the electronic conductivity within the film are intrinsically linked to variations in the oxygen stoichiometry. Additional examination of the influence of *p*O_2_ on the temporal evolution of σ_ac_ is shown in Figure S3 in the Supporting Information.

**Figure 4:**
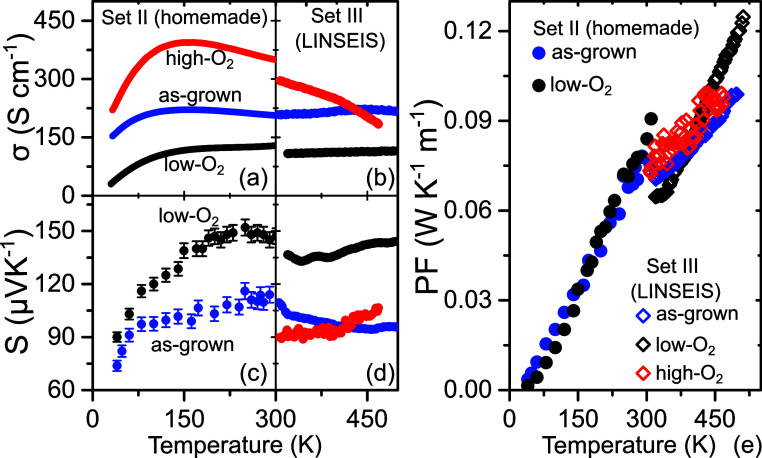
TE characterization of the as-grown (blue dots and lines), high (red dots and lines), and low (black dots and lines) O_2_-annealed films. Temperature dependence of the electrical conductivity (a,b), thermopower (c,d), and power factor (PF = *S*^2^σ*T*) of set II and set III films (e).

Figure 4 provides an overview of the temperature-dependent TE properties of set II and III samples spanning the range of 30–500 K. At a low temperature of 30–300 K (set II), the as-grown film exhibits metallic behavior of σ within the range of 330–150 K, followed by a downturn in σ below 150 K, a pattern reminiscent of observations in single crystals^14,32,55^ and thin films reported by others.^40,41^

The high-O_2_ film exhibited an enhanced σ up to ∼400 S cm^−1^, coupled with a temperature dependence similar to that of the as-grown film (see Figure 4a). The metallic σ–T behavior within the high-O_2_ film, spanning 300–150 K, and its pronounced downturn below 150 K suggest that the film approached near stoichiometry. In contrast, the σ value of the low-O_2_ film at 300 K exhibited a significant decrease, reaching approximately 130 S cm^−1^ compared to that of the as-grown film. Particularly, the σ–*T* curve of the low-O_2_ film revealed a nonmetallic behavior within the range of 300–150 K with distinct thermal activation-like characteristics from 30 to 150 and 150–300 K. This observation strongly indicates the high sensitivity of the film’s metallic character to variations in oxygen gas pressure and hence oxygen stoichiometry. An enhanced visualization of this behavior is displayed in Figure S3 in the Supporting Information.

Table 2 shows a comparative analysis of the relative changes in *R*_H_, *n*, and σ among the as-grown and annealed films at 300 K. It is evident that a higher *p*O_2_ during annealing corresponds to a higher σ, signifying that high-O_2_- and low-O_2_-annealed films possess higher and lower oxygen contents, respectively, in comparison to the as-grown film. This distinction is further corroborated by the *R*_H_ measured for each of the films at 300 K. The increase or decrease of σ might either be associated with the change of *n* or with the variation of electron mobility (*μ*). As can be seen in Table 2, the relative change of μ of the as-grown and annealed films is insignificant compared to the variation in *n*. Therefore, it can be concluded that the change of conductivity at 300 K is due to the change of variation in carrier density resulting from the deviation of oxygen content of the films.

**Table 2:** TE Parameters of the Films at Room Temperature^a^

sample	σ (S·cm^–1^)	*R*_H_ (10^–3^ cm^3^·C^–1^)	*n* (10^20^ cm^–3^)	*μ* (cm^2^·V^–1^·s^–1^)	*x*^b^ (e/unit cell)
low-O_2_	129	20.7 ± 1.4	3.0 ± 0.2	2.67 ± 0.2	0.11 ± 0.01
as-grown	206	11.4 ± 0.85	5.4 ± 0.4	2.38 ± 0.1	0.20 ± 0.01
high-O_2_	350	6.3 ± 0.40	9.9 ± 0.6	2.20 ± 0.09	0.37 ± 0.01

a The electron density (*n*) and mobility (μ) were estimated from the measured Hall coefficient (*R*_H_) and electronic conductivity (σ), respectively.

b The number of electrons per unit cells was calculated using the volume (*V*) of rock salt layer given by *V* = 372.3 Å^3^, where *a* = 4.9 Å, *b* = 5.1 Å, and *c* = 14.9 Å.^56^

Remarkably, the values of σ and S in films from set II (Figure 4a,c, respectively) and set III (Figure 4b,d, respectively) at 300 K, along with their temperature dependencies, exhibit a high degree of similarity, validating the reliability of thermopower measurements in both setups. Particularly, *S* displays a rapid increase between 40 and 100 K, transitioning to weak temperature dependence (or near-temperature independence) above 200 K. The measured power factor (PF) for BSCO films (Figure 4e) at 300 K is comparable to those reported in the existing literature.^40,57,58^ This good agreement, coupled with the fact that the bulk thermal conductivity of polycrystalline Bi_2_Sr_2_Co_2_O_*y*_ is temperature independent and fluctuates around 1.1 W·K^–1^·m^–1^ in the temperature range of 300–500 K,^16^ suggests a lower limit for ZT of approximately 0.06–0.1 within the same temperature range.

The weak temperature dependence of *S* at higher temperatures (300–500 K) is typical for the systems with localized charge carriers or polarons in a narrow band near-Fermi energy. When thermal energy (*k*_B_·*T*, where *k*_B_ is the Boltzmann constant) is higher than the bandwidth, then *S* is dominated by the entropy of the statistical distribution of the localized charge carriers over available sites.^47,59,60^ In the case of BSCO, the source of entropy is given by the distribution of Co^4+^ electrons over Co^3+^ sites, which can be mathematically expressed by modified Heike’s formula^61,62^ as follows
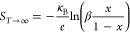
1where β and *x* are the ratio of spin–orbit degeneracy factor of Co^3+^ to Co^4+^ ions and the ratio of electrons over sites, respectively. Here, “*x*” can simply be expressed as electrons per unit cell.

**Figure 5:**
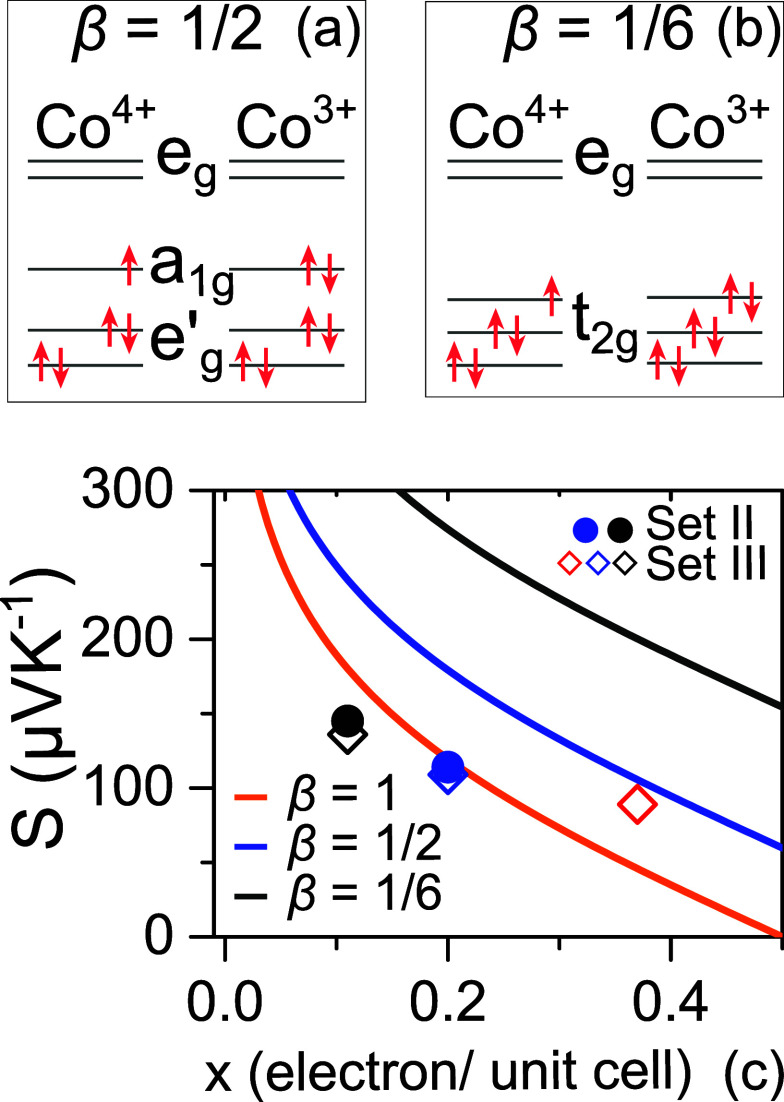
Schematic illustration of the atomic electronic states of Co^3+^ and Co^4+^ ions in Bi_2_Sr_2_Co_2_O_*y*_. (a) Spin states and orbital degeneracies of Co^3+^ and Co^4+^ ions in an octahedral crystal field and (b) lifting orbital degeneracy of Co^4+^ ions due to triangular distortion in the CoO_2_ layer of Bi_2_Sr_2_Co_2_O_*y*_. (c) Comparison of the expected and measured thermopower of BSCO sets II and III at 300 K. Solid blue and black dots are the measured thermopower of as-grown and low-O_2_ set II films at their corresponding “*x*” values at 300 K. Empty blue, black, and red diamonds correspond to thermopower at 300 K of as-grown, low- and high-O_2_-annealed films of set III. The solid lines indicate the expected thermopower obtained from eq 1 at different β values.

Evidently, the determination of *S* can be calculated by using eq 1 if β and *x* are known. The value of *x* may be estimated from the electron density measurements obtained via the Hall effect, computed by multiplying the electron density by the unit cell volume, as indicated in Table 1. Conversely, the calculation of β needs knowledge of the spin states of Co^3+^/Co^4+^ pairs and their associated orbital degeneracies. Specifically, the electronic configurations for low spin (LS) Co^3+^ and Co^4+^ ions are t_2g_^6^ e_g_^0^ and t_2g_^5^ e_g′_^0^, respectively. In the context of an octahedral crystal field splitting, the calculated β equals 1/6 (see Figure 5a). However, due to triangular distortions within the CoO_2_ layer, the t_2g_ energy states further split into a_1g_ and e_g_^′^ states.^12^ As a result, spin states do not change; rather, the orbital degeneracy is lifted,^51,63,64^ thereby yielding β = 1/2 (see Figure 5b).

Figure 5c shows the expected doping dependence of *S* (continuous lines) from eq 1 within *x* = 0–0.5 (i.e., up to 0–50% doping) for all possible β values (1, 1/2, and 1/6). The calculated values of *S* from the measured *x* (by using eq 1) and the measured *S* for the films at 300 K are shown as discrete data points. A reasonable agreement between the expected and measured *S* can be found around β = 1. This means that the spin–orbit degeneracy may not be an important factor which influences the thermopower of highly oriented misfit BSCO thin films.

## Conclusions

In this work, a series of highly *c*-axis-oriented layered misfit cobaltite Bi_2_Sr_2_Co_2_O_*y*_ thin films were grown by the PLD technique. We find that the metallic-like conductivity of these films is very sensitive to its oxygen nonstoichiometry, i.e., films with lower oxygen content exhibit thermal activation-like transport properties. A modulation of their TE properties was examined by the thermal annealing process under different gas pressures. The film annealed at lower oxygen pressure shows higher TE power factor. A maximum power factor multiplied by the temperature, PF ∼ 0.12 W m^–1^ K^–1^, was measured at *T* = 500 K. Our analysis indicates that the evaluation mechanism of thermopower in our BSCO films remains independent of the spin–orbit degeneracy factor at room temperature. This observation provides valuable insights into the underlying mechanisms that govern the TE properties of oxide materials, thereby facilitating the identification of novel high-performance materials.

## Supporting Information Available

Calibration curves for Seebeck measurements; photograph of the device; ac electrical conductivity measurements for different oxygen atmosphere; and Arrhenius-like plot of the experimental resistivity as a function of temperature (PDF)
