# Supplementary material for: Impact of Oxygen Stoichiometry on the Thermoelectric Properties of Bi2Sr2Co2Oy Thin Films
Source: ACS Appl Energy Mater. 2024 May 14;7(10):4504–12. doi: 10.1021/acsaem.4c00551 (PMC11134316; doi:10.1021/acsaem.4c00551)
Supplement: Supplementary file 1 — ae4c00551_si_001.pdf [file ae4c00551_si_001.pdf]

Impact of the oxygen stoichiometry on thermoelectric properties $Bi_2Sr_2Co_2O_y$ thin films

Arindom Chatterjee,[1] Alexandros El Sachat,[2] Clivia M. Sotomayor Torres+,[1,3] José Santiso[1] and Emigdio Chavez-Angel*[1]*

[1] Catalan Institute of Nanoscience and Nanotechnology (ICN2), CSIC, Barcelona Institute of Science and Technology (BIST), Bellaterra-08193, Spain

[2] Institute of Nanoscience and Nanotechnology, National Center for Scientific Research "Demokritos",15341 Athens, Greece

[3] ICREA—Catalan Institute for Research and Advanced Studies, 08010 Barcelona, Spain

*Corresponding author: Jose Santiso: jose.santiso@icn2.cat, Emigdio Chavez-Angel: emigdio.chavez@icn2.cat,

+Present address: International Iberian Nanotechnology Laboratory, Av. Mestre José Veiga s/n, 4715-330 Braga, Portugal

Keywords: Thermoelectrics, misfit cobaltates, oxygen annealing, $Bi_2Sr_2Co_2O_y$, spin-orbit degeneracy, power factor

Seebeck measurements and sample configuration

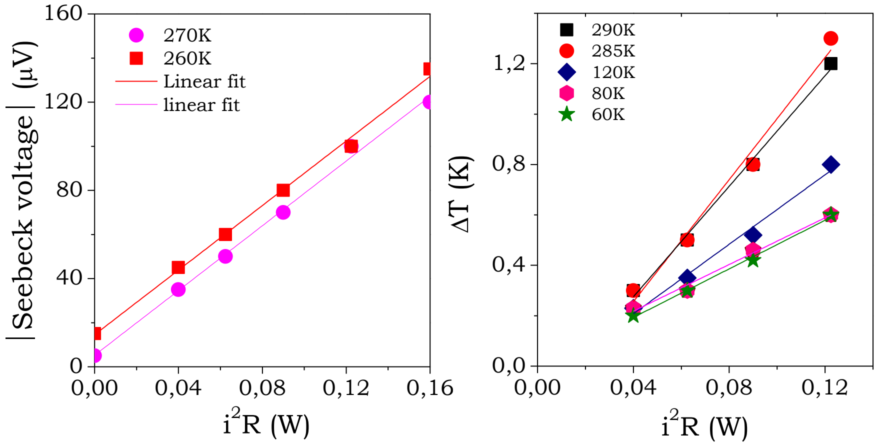

Figure S1. Temperature gradients and corresponding thermoelectric voltages of BSCO films at a fixed temperature varied linearly with the increasing power to the heater. Temperature gradient decreased with decreasing temperature at a fixed power to the heater. This is due to the fact that the thermal conductivity of $LaAlO_3$ substrates increases within 290-60 K[1].

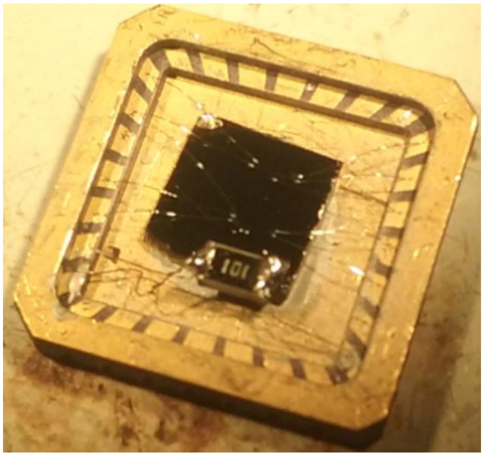

Figure S2. Photograph of the sample configuration for homemade Seebeck measurements.

ac Electrical conductivity measurements

Figure S3a shows the impact of the *p*O_2 on the temporal evolution of ac electrical conductivity measurements at T = 723 K. As *p*O_2 increases, a clear reduction in the response time (τ) of σ_{ac} is observed (**Figure S3b** to **d**), the differences in the time response reflect the increasing oxidation reaction kinetics with increasing *P*O_2.

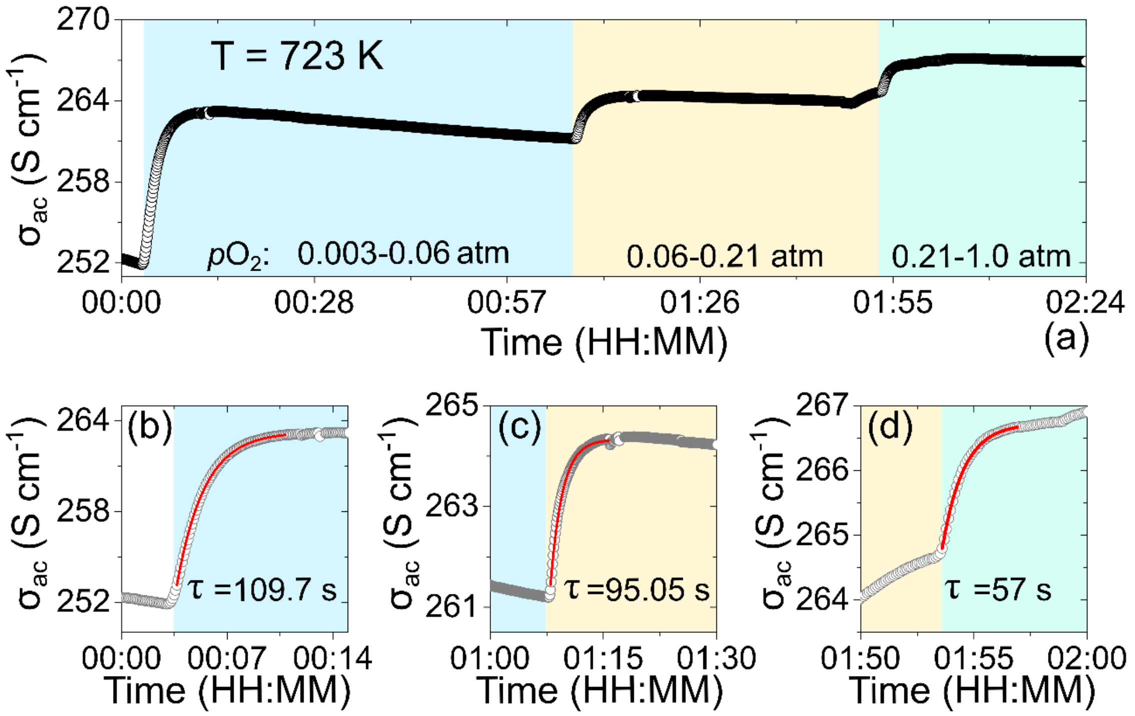

Figure S3. ac electrical conductivity as function of time at three *p*O_2 pressures **(a)** Full time-dependence evolution. Zoom around the **(b)**, **(c)** and **(d)**. The red line represents the exponential fit to estimate the response time (τ) of the σ_{ac} at each pressure.

Activation energy

Figure S4 shows Arrhenius-like plot of the experimental resistivity as function of temperature. The plot is based on the dependence of electrical conductivity with temperature based on the small polaron hopping model given by:[2]

$$\sigma(T) = \frac{\sigma_0}{T}\exp\left(-\frac{E_A}{k_B T}\right) \tag{1}$$

Activation-like energy (E_A) was derived from analyzing the slope of the natural logarithm of temperature-dependent resistivity multiplied by temperature (ln(σ·T)) plotted against the reciprocal of temperature (1/T) given by:

$$\ln(\sigma \cdot T) = -\frac{E_A}{k_B}\frac{1}{T} + \ln(\sigma_0) \tag{2}$$

where k_B is the Boltzmann's constant and σ_0 is a constant factor called the residual conductivity. Temperature regimes were selected through linear fitting, ensuring an R^2 coefficient > 0.998.

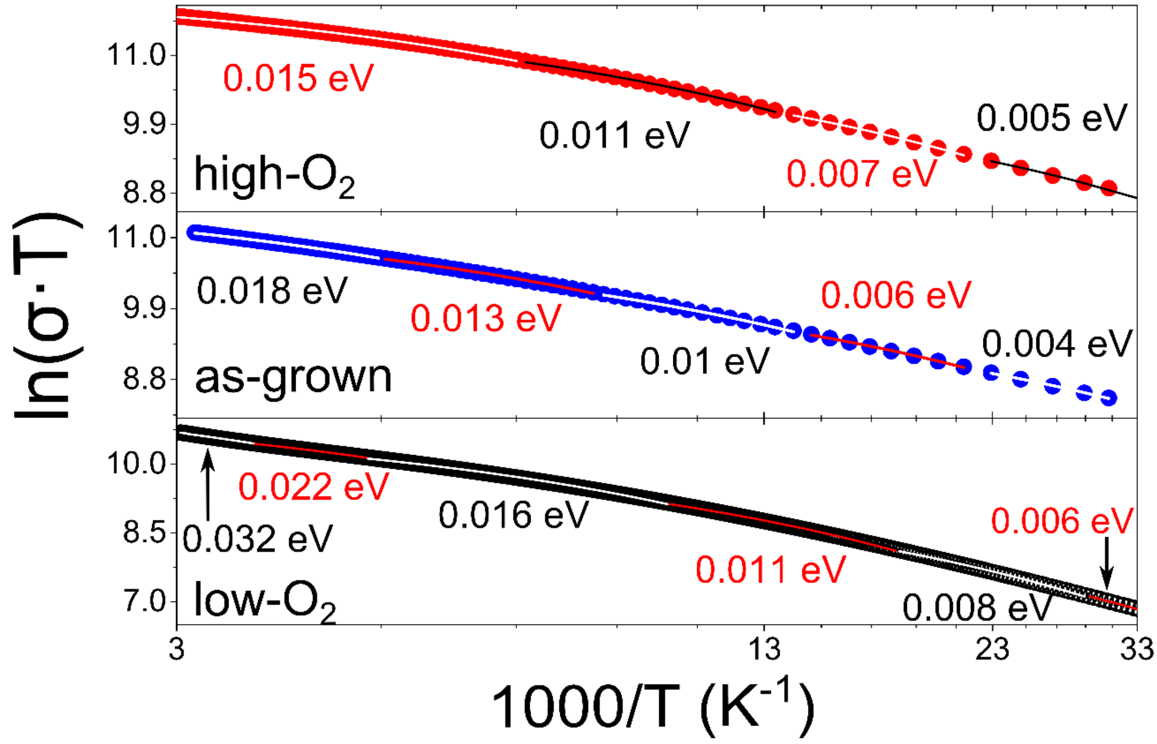

Figure S4. ln(σ·T) versus 1000/T for each sample. In each temperature range we can observe that each resistivity has its own slope indicating different activation-like energy.

References

(1) Langenberg, E.; Ferreiro-Vila, E.; Leborán, V.; Fumega, A. O.; Pardo, V.; Rivadulla, F. Analysis of the Temperature Dependence of the Thermal Conductivity of Insulating Single Crystal Oxides. *APL Mater.* **2016**, *4* (10), 104815. https://doi.org/10.1063/1.4966220.

(2) Hira, U.; Han, L.; Norrman, K.; Christensen, D. V.; Pryds, N.; Sher, F. High-Temperature Thermoelectric Properties of Na- and W-Doped $Ca_3Co_4O_9$ System. *RSC Adv.* **2018**, *8* (22), 12211–12221. https://doi.org/10.1039/C8RA01691G.
